# TP53 drives neuronal ferroptosis by promoting KLHL4-mediated SLC7A11 ubiquitination after spinal cord injury

**DOI:** 10.4103/NRR.NRR-D-24-01612

**Published:** 2025-09-03

**Authors:** Yu Kang, Qiangwei Li, Tianlun Zhao, Haojie Zhang, Yuejian Sun, Yilong Zhang, Da An, Zongsheng Yin, Yong Xuan, Peigen Xie

**Affiliations:** 1 Department of Spine Surgery, The Third Affiliated Hospital, Sun Yat-sen University, Guangzhou, Guangdong Province, China; 2 Department of Orthopedics, Zhaoqing Hospital, The Third Affiliated Hospital of Sun Yat-sen University, Zhaoqing, Guangdong Province, China; 3 Department of Orthopedics, The First Affiliated Hospital of Anhui Medical University, Hefei, Anhui Province, China; 4 School of Basic Medical Sciences, Anhui Medical University, Hefei, Anhui Province, China; 5 Department of Orthopedics, The Second People’s Hospital of Hefei, Hefei, Anhui Province, China

**Keywords:** bioinformatics, ferroptosis, glutathione peroxidase 4, Kelch-like protein 4, nerve regeneration, neuronal survival, SLC7A11, spinal cord injury, tumor protein 53, ubiquitination

## Abstract

Ferroptosis constitutes a pivotal pathological event following spinal cord injury and presents substantial challenges to the restoration of neurological function. Cystine-glutamate transporter SLC7A11 is essential for maintaining cellular redox homeostasis and resisting ferroptosis. However, the mechanisms underlying neuronal ferroptosis caused by SLC7A11 downregulation following spinal cord injury remain unclear. Herein, we provide evidence that tumor protein 53, a negative regulator of SLC7A11, was significantly upregulated post–spinal cord injury. Transcriptomic analysis indicated that tumor protein 53 was associated with injury severity. We subsequently confirmed that tumor protein 53 inhibition restored the expressions of SLC7A11 and glutathione peroxidase 4, alleviated neuronal ferroptosis, and improved neurological function in a contusion spinal cord injury rat model. The regulatory effects of tumor protein 53 on the transcription and ubiquitination of SLC7A11 were further elucidated using chromatin immunoprecipitation polymerase chain reaction and cleavage under targets and tagmentation techniques. Additionally, Kelch-like protein 4, an E3 ubiquitin ligase adaptor, was demonstrated to play an important role in the tumor protein 53-mediated ubiquitination of SLC7A11. In summary, the present study elucidated the possible mechanisms of tumor protein 53–mediated neuronal ferroptosis in spinal cord injury, thereby providing potential targets and insights for clinical translation.

## Graphical abstract

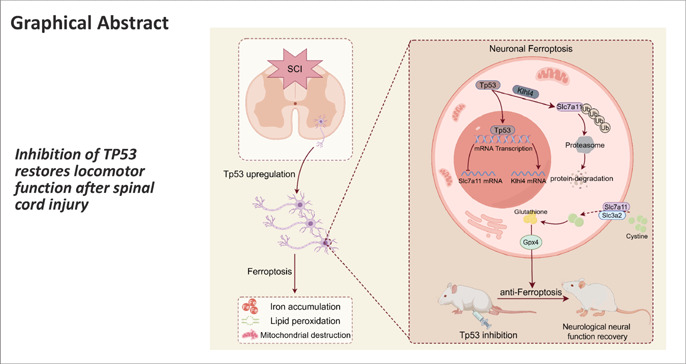

## Introduction

Spinal cord injury (SCI) constitutes a severe form of central nervous system damage; it frequently results in the loss of sensory and motor functions below the injury level (Liu et al., 2022). SCI places substantial economic and psychological strains on society and families because of high disability rates and treatment challenges (Pang et al., 2022; Ke et al., 2023). Consequently, it is important to identify potential therapeutic targets by exploring the pathological mechanisms of SCI.

After SCI, there are many challenges to neuronal survival, including inflammation, ischemia, edema, and oxidative stress (Zhang et al., 2023b). Damage to the blood–spinal cord barrier contributes to the development of a microenvironment with localized iron overload and reactive oxygen species (ROS) accumulation, meaning that ferroptosis represents a predominant form of neuronal death (Kang et al., 2023; Xiong et al., 2023). Ferroptosis is increasingly recognized as a critical pathological event in secondary SCI (Li et al., 2023), and is characterized by iron overload and the accumulation of ROS and malondialdehyde (MDA; an indicator of lipid peroxidation) (Lin et al., 2022b; Demir et al., 2022). Compared with primary SCI, secondary SCI exhibits a higher degree of reversibility (Xu et al., 2023). There have thus been increasing efforts to treat SCI by regulating ferroptosis, encompassing ferroptosis inhibitors, hyperbaric oxygen therapy, stem cell therapy, and biomaterials (Yao et al., 2019, 2023a, b; Geng et al., 2023). Although targeting ferroptosis for SCI treatment shows promise, the molecular mechanisms underlying SCI-induced neuronal ferroptosis remain to be comprehensively elucidated.

Tumor protein 53 (TP53) is a multifunctional molecule that participates in the regulation of various biological processes, including the cell cycle, DNA repair, and apoptosis (Xu et al., 2013). TP53 triggers ferroptosis via both glutathione (GSH)-dependent (Jiang et al., 2019) and -independent pathways (Chu et al., 2019). TP53 also reportedly mediates ferroptosis in cancer cells by negatively regulating cystine-glutamate transporter Slc7a11 transcription (Jiang et al., 2015). The ubiquitination of SLC7A11 is a key factor in the progression of ferroptosis (Zhou et al., 2024); however, the regulatory effects of TP53 on SLC7A11 ubiquitination remain unclear.

As a member of the Kelch-like protein (KLHL) family, KLHL4 contains a BTB/POZ domain, a BACK domain, and Kelch repeats (Dhanoa et al., 2013), and plays a critical role in the ubiquitination process. The BTB/POZ and BACK domains interact with Cullin-3 and RING-box protein 1 to inform the Cullin3–RING E3 ubiquitin ligase complexes, and the Kelch-repeat domain is responsible for the recognition and binding of substrates (Yumimoto et al., 2023). Nonetheless, it has not yet been investigated whether the KLHL4-mediated ubiquitination process exerts any effects in SCI.

Ferroptosis contributes to neuronal loss after SCI. The present study therefore integrated bioinformatic analysis with *in vivo* and *in vitro* experiments to elucidate the molecular mechanisms underlying SCI-induced neuronal ferroptosis. We aimed to revealed the regulatory role of TP53 in neuronal ferroptosis, and hypothesized that inhibiting TP53 expression may enhance neurological recovery following SCI. Our findings not only advance the understanding of neuronal ferroptosis in SCI but also provide potential targets for clinical translation.

## Methods

### Animals

Compared with female rats, male rats exhibit a higher susceptibility to hematuria and urinary complications after SCI (Ferrero et al., 2015). Female rats are therefore used most frequently in SCI-associated studies. We purchased 216 adult female Sprague Dawley rats (aged 6–8 weeks, weighing 180–220 g) from the Animal Center of Anhui Medical University (license No. SYXK (Wan) 2017-001) and from Guangzhou Ruige Biological Technology Co., Ltd. (license No. SCXK (Yue) 2023-0059). The animals were housed under standardized conditions, which included a 12-hour light/dark cycle, a controlled temperature of 25 ± 3°C, and humidity levels of 60%–70%. All animal experiments and procedures were conducted in accordance with the National Institutes of Health Guide for the Care and Use of Laboratory Animals. The study received ethical approval from the Ethics Committee of Anhui Medical University (approval No. LLSC 20201135, approved January 21, 2021) and Seyotin Biotechnology Co., Ltd. (approval No. SYT20230145, approved December 15, 2023).

Pifithrin-α (PFT-α; S1816, Beyotime, Shanghai, China) is a well-established TP53 inhibitor. It was used to investigate the effects of TP53 on neurological function recovery and neuronal ferroptosis after SCI. First, the inhibitory efficiency of PFT-α on TP53 was assessed. Rats were randomly divided into six groups (five rats per group): Sham, SCI, SCI + vehicle, SCI + PFT-α (0.5 mg/kg, intraperitoneal injection), SCI + PFT-α (1 mg/kg), and SCI + PFT-α (5 mg/kg) groups.

In the behavior and photothermal sensitivity tests, rats were randomly divided into four groups (five rats per group): Sham, SCI, SCI + low-dose PFT-α (0.5 mg/kg), and SCI + high-dose PFT-α (1 mg/kg) groups. In the molecular biological tests, rats were randomized to Sham, SCI, and SCI + PFT-α (1 mg/kg) groups (five rats per group).

### Spinal cord injury model

To establish the SCI model, rats were anesthetized via intraperitoneal injection of 2% sodium pentobarbital (30 mg/kg, Sigma-Aldrich/Merck, Darmstadt, Germany) before applying a modified Allen’s method. A T10 laminectomy was performed to expose the spinal cord, which was then contused using an impactor (10 g weight dropped from a height of 5 cm) (Kang et al., 2022). The Sham group underwent T10 laminectomy only, without contusion. Postoperatively, rats received intramuscular injections of penicillin (to prevent infection) and carprofen (for pain relief) for the first 3 consecutive days. Additionally, to aid in bladder voiding, the rats were manually stimulated by gentle squeezing once daily until euthanasia.

### Tissue RNA sequencing

At 3 and 14 days post-injury (dpi), 5-mm sections of spinal cord tissue, including the lesion epicenter, were collected for RNA sequencing (RNA-seq) analysis (*n* = 3), which was conducted by Bioprofile Technology Co., Ltd. (Shanghai, China). Following RNA extraction, purification, and library construction, the libraries were sequenced using next-generation sequencing on the Illumina HiSeq platform with paired-end reads (Illumina, San Diego, CA, USA). The resulting raw data were filtered to obtain high-quality sequences, which were then aligned to the rat reference genome. Gene expression levels were quantified from the alignment results.

### Bioinformatics

A single-cell RNA-seq (scRNA-seq) dataset (GSE213240, https://www.ncbi.nlm.nih.gov/geo/query/acc.cgi?acc=GSE213240) and two bulk RNA-seq datasets (GSE45006 for compression SCI, https://www.ncbi.nlm.nih.gov/geo/query/acc.cgi?acc=GSE45006; GSE29488 for transection SCI, https://www.ncbi.nlm.nih.gov/geo/query/acc.cgi?acc=GSE29488) were downloaded from the Gene Expression Omnibus database (Clough et al., 2024). The GSE213240 dataset comprised SCI rat samples characterized by three different severity levels: mild, moderate, and severe. Together with datasets GSE45006, GSE29488, and the transcriptomic data from our study, this constituted a bulk RNA-seq dataset that captured a spectrum of injury severity. The Seurat package (https://satijalab.org/seurat/articles/install_v5.html) was used for scRNA-seq data processing. Protein–protein interaction analysis was performed using the STRING database (http://string-db.org) and Cytoscape software v3.7.2 (www.cytoscape.org). Hubgene expression was visualized using the FeaturePlot function (within the Seurat package). The Weighted Gene Co-expression Network Analysis package (WGCNA, http://www.genetics.ucla.edu/labs/horvath/CoexpressionNetwork/Rpackages/WGCNA) was used for bulk RNA-seq data processing following normalization. A gene set related to ferroptosis was collected from FerrDb v2.0 (http://www.zhounan.org/ferrdb/current/) (Zhou et al., 2023), and a gene set related to ubiquitination was collected from iUUCD v2.0 (http://www.iuucd.biocuckoo.org/) (Zhou et al., 2018). Subsequent bioinformatic analyses, including differential gene expression and Gene Ontology enrichment analyses, were conducted using R software (version 4.2.2, https://www.r-project.org/). Visualization of the bioinformatic results was performed using an online platform for data analysis and visualization (available at https://www.bioinformatics.com.cn) (Tang et al., 2023).

### Western blotting

Lysis of the isolated spinal cords (*n* = 5) or cells (*n* = 3) was performed on ice using radio-immunoprecipitation assay lysis buffer (RIPA; P0013B, Beyotime) supplemented with 1% phenylmethanesulfonyl fluoride (PMSF). Subsequently, the lysates were centrifuged at 4°C at a speed of 12,000 × *g* for 25 minutes. The supernatant was collected for protein quantification using an enhanced Bicinchoninic Acid Protein Assay Kit (P0009, Beyotime). Samples were mixed with 5× loading buffer (4:1; P0015L, Beyotime), denatured at 100°C for 10 minutes, separated with 10% sodium dodecyl sulfate (SDS)–polyacrylamide gel electrophoresis (PG112, Epizyme Biotech, Shanghai, China), and transferred to polyvinylidene fluoride membranes (Millipore, Burlington, MA, USA). Membranes were then blocked in Tris-buffered saline with Tween 20 solution (PS103, Epizyme Biotech) containing 5% non-fat milk at 25°C for 2 hours, followed by incubation overnight at 4°C with primary antibodies. After two 15-minute washes with Tris-buffered saline with Tween 20, membranes were incubated with horseradish peroxidase-conjugated secondary antibodies for 2 hours at 25°C. Detailed information about the primary and secondary antibodies used is provided in **Additional Table 1**. Protein bands were visualized using the Tanon-5200CE imaging system (Tanon, Shanghai, China) and an enhanced chemiluminescence system (SQ101, Epizyme Biotech). Quantitation of band intensities was performed using ImageJ v1.53c (National Institutes of Health, Bethesda, MD, USA). Glyceraldehyde 3-phosphate dehydrogenase was used as the internal reference.

**Additional Table 1:** Antibodies utilized for western blotting

Name	Animal species	Cat#	RRID	Dilution	Supplier
Anti-TP53	Rabbit	TA0879	NA	1:1000	Abmart, Shanghai, China
Anti-PTGS2	Rabbit	T58852	AB_2936469	1:1000	Abmart
Anti-SLC7A11	Rabbit	T57046	AB_2936462	1:1000	Abmart
Anti-GPX4	Rabbit	T56959	AB_2936461	1:1000	Abmart
Anti-Ubiquitin	Rabbit	T55965	AB_2936388	1:1000	Abmart
Anti-4HNE	Rabbit	ab46545	AB_722490	1:3000	Abcam,
Anti-GAPDH	Mouse	60004-1-Ig	AB_2107436	1:5000	Cambridge, UK Proteintech, Wuhan, China
HRP-conjugated goat anti-rabbit IgG(H+L)		SA00001-2	AB_2722564	1:10000	Proteintech
HRP-conjugated goat anti-mouse IgG(H+L)		SA00001-1	AB_2722565	1:10000	Proteintech

4HNE: 4-Hydroxynonenal; GAPDH: glyceraldehyde 3-phosphate dehydrogenase; GPX4: glutathione peroxidase 4; HRP: horseradish peroxidase; NeuN: neuronal nuclei; TP53: tumor protein 53.

### Immunohistochemical staining

At 14 dpi, rats (*n* = 5) were anesthetized and transcardially perfused with pre-cooled phosphate-buffered saline (PBS), followed by fixation with 4% paraformaldehyde. Spinal cord tissue was harvested and fixed in 4% paraformaldehyde for 24 hours. Following dehydration and paraffin embedding, 4-μm sections were cut using a microtome (Leica, Dresden, Germany; RM2255). After deparaffinization and rehydration, citrate buffer was used for antigen retrieval via the microwave thermal repair method. Next, spinal cord sections were incubated with 3% H_2_O_2_ for 15 minutes to inactivate endogenous peroxidases, and were then blocked with QuickBlock^TM^ Blocking Buffer (P0260, Beyotime) for 30 minutes. Sections were incubated overnight at 4°C with primary antibody against TP53 (mouse, 1:500, Cell Signaling Technology, Beverly, MA, USA; Cat# 2524, RRID: AB_331743) diluted in QuickBlock™ Primary Antibody Dilution Buffer (P0262, Beyotime). Following incubation with horseradish peroxidase-conjugated secondary antibody (anti-mouse, 1:500, Thermo Fisher Scientific, Waltham, MA, USA; Cat# 31430, RRID: AB_228307) for 45 minutes 25°C, the sections were visualized using a 3,3-Diaminobenzidine Horseradish Peroxidase Color Development kit (P0202, Beyotime). The stained sections were then observed and imaged using a Leica ICC50 W light microscope (Leica Microsystems Inc., Buffalo Grove, IL, USA). Quantification of immunohistochemical images was conducted using ImageJ v1.53c.

### Motor evoked potential detection

At 28 dpi, rats were anesthetized (*n* = 5), and the cerebral cortex below the bregma was exposed via a 3.0 mm × 3.0 mm craniotomy. The sciatic nerve was exposed near the greater trochanter of the femur. Bipolar electrodes were inserted into the cortex to a depth of 2.0 mm for stimulation, and the recording electrode was positioned in the contralateral sciatic nerve. A ground wire was placed between the stimulation and recording sites. Motor evoked potential (MEP) analysis was performed using the BL-420 Data Acquisition Analysis System (TECHMAN SOFT, Chengdu, China) and TM_WAVE biological information acquisition and analysis software. The parameters were set in accordance with those used in our previous study: amplifier range, 500 mV; time constant, direct current; filter, 30 kHz; and scan speed, 6.25 ms/division. The stimulation settings included single stimulus mode with a wave width of 1 ms, frequency of 10 Hz, and strength of 10 V.

### Behavioral assessments

Behavioral assessments were conducted using the footprint analysis; Basso, Beattie, and Bresnahan (BBB) scale; oblique board test; and swimming test (*n* = 5). In the footprint analysis, the fore and hind paws of rats were marked with non-toxic red and blue paint, respectively. Rats were guided to walk straight over a blank paper. Only trials in which rats maintained a continuous and direct trajectory were included in the analysis. Stride length—the distance between two successive rear paw prints—was then measured for quantitative analysis. The BBB scale, which ranges from 0 (indicating complete hind-limb disability) to 21 points (indicating normal locomotor function) (Basso et al., 1995), was used to evaluate locomotor function for 4 weeks following SCI surgery. Assessments were performed for each of the five rats across the different experimental groups at 1, 3, 7, 14, 21, and 28 dpi. At 28 dpi, the oblique board and swimming tests were administered. For the oblique board test (Zhu et al., 2024), rats were positioned on an inclined plane whose height progressively increased. The maximum angle at which the rats were able to maintain their position for a duration > 5 s was recorded. The swimming test was conducted in accordance with the Louisville Swim Scale (Smith et al., 2006), in which scores of 0–5, 6–11, and 12–17 correspond to poor, intermediate, and good performance levels, respectively. Each assessment was conducted by two researchers who were blinded to animal groups and time points, thereby ensuring an unbiased and standardized evaluation of hind-limb mobility post-SCI.

### Photothermal sensitivity test

Four weeks post-SCI, hind-limb sensitivity to photothermal stimulation was assessed using the Heat Pain Detector PL-200 (TECHMAN SOFT) (*n* = 5). Prior to evaluation, each rat was acclimated in the observation chamber for 30 minutes. Photothermal stimulation was administered via a heat source radiator with the output energy calibrated to 50%, targeting the feet of rats. The duration from stimulation onset to the foot withdrawal response was recorded for each rat.

### Magnetic resonance imaging

Magnetic resonance imaging was used to assess the spinal cord at 28 dpi, using a 3.0-Tesla MR system (Discovery MR750w, GE Healthcare, Chicago, IL, USA) equipped with a rat whole-body coil. Sagittal T2-weighted and T2 fat-suppressed images were acquired using the following parameters: repetition time = 4079 ms; echo time = 21 ms; field of view = 10 cm × 8 cm; and slice thickness = 1.5 mm (Kang et al., 2023).

### Tissue iron content assay

At 14 dpi, rats were anesthetized and perfused with pre-cooled sterile saline to remove erythrocytes from the spinal cords (*n* = 5). The injured tissue was then transferred to a sterile glass dish, and the spinal dura mater and nerve roots were carefully dissected. The tissues were homogenized in nine times their volume (volume/weight) of normal saline. After centrifugation at 600 × *g* for 10 minutes, the supernatant was collected and analyzed using a tissue iron assay kit (A039-2-1, Nanjing Jiancheng Bioengineering Institute, Nanjing, China). The optical density was measured at 520 nm.

### Histological staining

Spinal cord sections were subjected to histological staining, comprising hematoxylin and eosin (HE), Nissl, and Prussian blue staining, at 14 dpi (*n* = 5). Paraffin-embedded tissue sections were obtained in accordance with the protocol described in the Immunohistochemical staining section. The sections then underwent histological staining using an HE Stain Kit (G1120, Solarbio, Beijing, China), Nissl Stain Kit (G1430, Solarbio), and Prussian Blue Iron Stain Kit (enhanced with 3,3′-diaminobenzidine) (G1428, Solarbio). The stained sections were then observed and imaged using a Leica ICC50 W light microscope. Quantitative analyses of HE and Nissl staining were performed using ImageJ software (version 1.53c) by comparing the cavity area and number of Nissl^+^ neurons across different groups.

### FerroOrange staining

At 14 dpi, rats (*n* = 5) were anesthetized and transcardially perfused with pre-cooled PBS, followed by fixation with 4% paraformaldehyde. Rat tissue was then subjected to a sequential dehydration process using sucrose solutions of progressively increasing concentrations (10%, 20%, and 30% in 0.1 M PBS). The specimens were embedded in optimal cutting temperature compound (Sakura Finetek, Tokyo, Japan; Cat# 4583) and sectioned at 10 μm thickness using a low-temperature thermostatic microtome (Leica, CM1950). Following three 5-minute washes with PBS containing 0.1% Tween 20 (PBST), the sections were incubated with 1 μM FerroOrange (DOJINDO, Kumamoto, Japan; F374) at 37°C for 30 minutes. For *in vitro* experiments (*n* = 3), PC12 cells were seeded in confocal dishes (801001, NEST, Wuxi, China) and co-incubated with 1 μM FerroOrange probe at 37°C for 30 minutes. Sections and dishes were observed and imaged using a Leica STELLARIS STED confocal microscope (Leica). Quantitative analysis of FerroOrange staining was performed using ImageJ software (version 1.53c) by comparing the fluorescent area across different groups.

### Liperfluo staining

At 14 dpi, rats (*n* = 5) were anesthetized and transcardially perfused with pre-cooled PBS, followed by fixation with 4% paraformaldehyde. Frozen tissue sections were collected as described in the FerroOrange staining section. Tissue sections were then incubated with 1 μM Liperfluo (L248, DOJINDO) at 37°C for 40 minutes. Following three 5-minute washes with PBST, sections were observed and imaged using a Leica STELLARIS STED confocal microscope. Quantitative analysis of Liperfluo staining was performed using ImageJ software (version 1.53c) by comparing the mean fluorescence intensity across different groups.

### Malondialdehyde content assay

Tissue samples from the lesion epicenter were harvested at 14 dpi and subsequently lysed in a nine-fold volume-to-weight ratio of lysis buffer (P0013, Beyotime) (*n* = 5). For cell samples (*n* = 3), 0.1 mL of lysis buffer was used per 1 million cells. The lysed samples were centrifuged at 12,000 × *g* for 10 minutes, and supernatants were carefully collected for subsequent analysis. MDA levels were determined using the Lipid Peroxidation MDA Assay Kit (S0131M, Beyotime) in accordance with the manufacturer’s instructions.

### Immunofluorescence staining

At 14 dpi, rats (*n* = 5) were anesthetized and transcardially perfused with pre-cooled PBS, followed by fixation with 4% paraformaldehyde. Paraffin-embedded and frozen tissue sections were collected as described in previous sections. The paraffin sections underwent antigen retrieval followed by blocking with QuickBlock Blocking Buffer for 30 minutes at 25°C. The frozen sections were washed with PBST and subsequently subjected to a blocking step. Next, sections were incubated overnight with primary antibodies diluted in QuickBlock Primary Antibody Dilution Buffer at 4°C. The following day, sections underwent three 10-minute washes with PBST before being incubated with secondary antibodies diluted in Immunol Fluorescence Staining Secondary Antibody Dilution Buffer (Beyotime, P0108) for 1.5 hours in a wet box at 37°C. Post-incubation, the sections were washed with PBST three times for 10 minutes each. Antifade mounting medium containing 4′,6-diamidino-2-phenylindole (G1407, Servicebio, Wuhan, China) was used to seal the sections.

For cellular experiments (*n* = 3), PC12 cells were seeded in 24-well culture plates with 14-mm cell-climbing slices (BS-14-RC, Biosharp, China). The cell-climbing slices were then fixed with 4% paraformaldehyde for 15 minutes. Subsequently, the cell-climbing slices were subjected to a staining protocol similar to that used for immunofluorescence staining of tissue section. Detailed information about the primary and secondary antibodies used is provided in **Additional Table 2**. Images were captured using an Axio Imager Z2 fluorescence microscope (Zeiss, Oberkochen, Germany) and a Leica STELLARIS STED confocal microscope.

**Additional Table 2:** Antibodies utilized for immunofluorescence staining

Name	Animal species	Cat#	RRID	Dilution	Supplier
Anti-4HNE	Rabbit	ab46545	AB_722490	1:1000	Abcam, Cambridge, UK	
Anti-NF200	Chicken	ab4680	AB_304560	1:500	Abcam	
Anti-GFAP	Rabbit	80788	AB_2799963	1:500	Cell Signaling Technology, Danvers, MA, USA	
Anti-PSD95	Rabbit	3450	AB_2292883	1:500	Cell Signaling Technology	
Anti-SYN	Mouse	67864-1-Ig	AB_2918622	1:200	Proteintech	
Anti-NEUN	Mouse	ab104224	AB_10711040	1:1000	Abcam	
Anti-NEUN	Rabbit	ab177487	AB_2532109	1:1000	Abcam	
Anti-NEUN	Rat	ab279297	AB_3095692	1:500	Abcam	
Anti-VSX2	Sheep	ab16141	AB_302278	1:200	Abcam	
Anti-SLC7A11	Rabbit	26864-1-AP	AB_2880661	1:500	Proteintech	
Anti-SLC7A11	Mouse	98051	AB_2800296	1:500	Cell Signaling Technology	
Anti-GPX4	Mouse	67763-1-Ig	AB_2909469	1:500	Proteintech	
Anti-TP53	Mouse	2524	AB_331743	1:500	Cell Signaling Technology	
Anti-BrdU	Mouse	66241-1-Ig	AB_2881630	1:200	Proteintech	
Anti-KLHL4	Rabbit	21191-1-AP	AB_10755294	1:500	Proteintech	
Goat anti-rabbit FITC		ZF-0311	AB_2571576	1:200	ZSGB-BIO, Beijing, China	
Goat anti-mouse FITC		ZF-0312	AB_2716306	1:200	ZSGB-BIO	
Goat anti-rabbit TRITC		ZF-0316	AB_2728778	1:200	ZSGB-BIO	
Goat anti-mouse TRITC		ZF-0313	AB_2571577	1:200	ZSGB-BIO	
Goat anti-chicken Alexa Fluor® 647	Goat	ab150175	AB_2732800	1:500	Abcam	
Goat anti-rabbit Alexa Fluor® 488	Goat	ab150077	AB_2630356	1:500	Abcam	
Goat anti-mouse Alexa Fluor® 594	Goat	ab150116	AB_2650601	1:500	Abcam	
Donkey anti-sheep Alexa Fluor® 555	Donkey	ab150178	NA	1:500	Abcam	

4HNE: 4-Hydroxynonenal; BrdU: 5-bromo-2'-deoxyuridine; FITC: fluorescein isothiocyanate; GFAP: glial fibrillary acidic protein; GPX4: glutathione peroxidase 4; KLHL: Kelch-like protein; NeuN: neuronal nuclei; NF200: neurofilament 200; PSD95: postsynaptic density protein 95; SYN: synaptophysin; TP53: tumor protein 53; TRITC: tetramethylrhodamine isothiocyanate; VSX2: visual system homeobox 2.

Quantitative analysis of immunofluorescence staining was performed using ImageJ software (version 1.53c) by comparing the mean fluorescence intensity or the fluorescent area across different groups. Co-localization analyses were conducted using the plot profile plugin within ImageJ software (version 1.53c).

### Transmission electron microscopy

Transmission electron microscopy (TEM) was performed as described previously (Kang et al., 2023). At 14 dpi, rats were anesthetized and perfused with 4% paraformaldehyde. The spinal cords from the lesion epicenter were isolated and cut into 1 mm3 pieces. For *in vitro* experiments, PC12 cells were collected using a cell scraper before being centrifuged at 250 × *g* for 5 minutes. The tissue pieces and cell pellets were then immersed in a fixative solution (P885738, Macklin, Shanghai, China) of 2% paraformaldehyde and 2.5% glutaraldehyde in PBS for 24 hours. Following phosphate buffer washes, the samples were fixed in phosphate buffer containing 1% OsO_4_ for 2 hours at 4°C, and were then thoroughly rinsed with double-distilled H_2_O. Next, the samples underwent staining with 2% aqueous uranyl acetate (Polysciences, Warrington, PA, USA) for 2 hours, followed by serial dehydration in 50%, 70%, 90%, and 100% ethanol, and 100% acetone. Samples were then embedded in epoxy resin. Ultrathin sections (70–90 nm) were cut using an ultramicrotome (EM UC7, Leica) and stained with lead citrate. The sections were observed and imaged using TEM (Talos L120C G2, Thermo Fisher Scientific).

### 5-Bromo-2′-deoxyuridine incorporation

To ascertain the origin of neurons in proximity to the injury site, the immunofluorescence of 5-bromo-2′-deoxyuridine (BrdU) and neuronal nuclei (NeuN) was analyzed. Within 2 weeks after modeling, rats received an intraperitoneal injection of BrdU (Sigma-Aldrich/Merck, Cat# 59-14-3) dissolved in saline (50 mg/kg) every 2 day. Before immunofluorescence staining, the sections were incubated with 2 M HCl for 30 minutes at 25°C to denature DNA. Subsequently, the BrdU signal was visualized by immunostaining procedures.

### Quantitative reverse transcription polymerase chain reaction

After being lysed in TRIzol reagent (1 mL per 100 mg of tissue), total RNA from spinal cord tissue (14 dpi) was extracted using chloroform, precipitated with isopropyl alcohol, and rinsed with 75% ethanol. Total RNA from PC12 cells was extracted using the SPARKeasy Cell RNA Kit (AC0205, SparkJade, Jinan, China) according to the manufacturer’s protocol. RNA purity and concentration were determined using the NanoDrop One Spectrophotometer (Thermo Fisher Scientific). For complementary DNA (cDNA) synthesis, the Evo M-MLV RT Master Mix (AG11706, Accurate Biotechnology, Beijing, China) was used following the manufacturer’s instructions to convert 500 ng of RNA into cDNA. The reaction conditions were as follows: an initial incubation at 37°C for 15 minutes, a brief exposure to 85°C for 5 seconds, and a final step at 4°C. Quantitative reverse transcription polymerase chain reaction (qRT-PCR) was performed using a LightCycler 96 system (Roche, Alameda, CA, USA) in a reaction volume of 20 μL. The reaction mixture consisted of 10 μL of 2× SYBR Green Pro Taq HS Premix (AG11701, Accurate Biotechnology), 70 ng of cDNA, 0.4 μL each of forward and reverse primers (**Additional Table 3**), and RNase-free water to a final volume of 20 μL. Two vice-holes were used in each sample, and β*-actin* was used as an internal reference gene for data normalization. The relative expression levels of the target genes were quantified using the 2^–ΔΔCT^ method (Livak and Schmittgen, 2001).

**Additional Table 3:** Primers used for quantitative reverse transcription-polymerase chain reaction

Gene	Sequence
*Ptgs2*	Forward: 5ʹ-CACGGACTTGCTCACTTTG-3ʹ Reverse: 5ʹ-AGCGTTTGCGGTACTCATT-3ʹ
*Slc7a11*	Forward: 5ʹ-GTTCAGACGATTGTCAGACAGAA-3ʹ Reverse: 5ʹ-ACCAATTCCTTTAGCCCATCA-3ʹ
*Klhl4*	Forward: 5ʹ-TACTGTCGCCTCAGTTGCTT-3ʹ Reverse: 5ʹ-AGCATAAAGTGCCCCCACAG-3ʹ
*β-actin*	Forward: 5ʹ-CACCATGTACCCAGGCATTG-3ʹ Reverse: 5ʹ-CCTGCTTGCTGATCCACATC-3ʹ

### Cell culture and viability assay

The PC12 cell line has been identified as a suitable *in vitro* model for investigating the biomedical characteristics of neurons (Liu et al., 2020). Additionally, the HEK293 cell line was selected because of its superior transfection efficiency and protein expression capability (Abegglen et al., 2015). The PC12 (China Center for Type Culture Collection, Wuhan, China; Cat# GDC0006, RRID: CVCL_0481) and HEK293 (China Center for Type Culture Collection, Cat# GDC0067, RRID: CVCL_0045) cell lines were cultured in Roswell Park Memorial Institute 1640 medium (RPMI-1640, Gibco, Carlsbad, CA, USA) supplemented with 10% fetal bovine serum (Gibco) and 1% penicillin-streptomycin (Beyotime) under conditions of 37°C and 5% CO_2_. Cell viability was assessed using the Cell Counting Kit-8 (C0037, Beyotime) in strict accordance with the manufacturer’s instructions. Each detection was conducted in triplicate for the *in vitro* experiments (*n* = 3 per group).

### Reagents, small interfering RNA, and plasmids for cell intervention

PC12 cells were used to establish a cell model of SCI-induced ferroptosis by treatment with H_2_O_2_ (200 μM) and Fe (20 μM) for 24 hours. The feasibility of the cell model was validated using a ferroptosis inducer (erastin, HY-15763, MCE, Shanghai, China) and inhibitor (ferrostatin-1, HY-100579, MCE). In this experiment, PC12 cells were grouped into control, H_2_O_2_ + Fe, Erastin (0.75 μM), and H_2_O_2_ + Fe + Ferrostatin-1 (2 μM) groups.

Small interfering RNA (siRNA) and overexpression (O/E) plasmids were used to regulate gene expression *in vitro*. To investigate the regulatory mechanism of TP53 on neuronal ferroptosis, we performed *Tp53* knockdown and O/E in the aforementioned cell model. PC12 cells were grouped into control, H_2_O_2_ + Fe, H_2_O_2_ + Fe + siRNA-*Tp53*, and H_2_O_2_ + Fe + O/E-*Tp53* groups.

To clarify the pathway of TP53-mediated SLC7A11 exhaustion, cycloheximide (CHX; a protein synthesis inhibitor, MCE, HY-12320), bafilomycin A1 (Baf-A1; an autophagosome-lysosome pathway blocker, MCE, HY-100558), and MG132 (a potent proteasome inhibitor, MCE, HY-13259) were used to treat PC12 cells in the presence and absence of TP53 O/E. PC12 cells were grouped into vector + CHX (50 μg/mL), O/E-*Tp53* + CHX (50 μg/mL), O/E-*Tp53* + Baf-A1 (1 μM) + CHX (50 μg/mL), and O/E-*Tp53* + MG132 (10 μM) + CHX (50 μg/mL) groups. The half-life of SLC7A11 was calculated using GraphPad Prism software (version 8.0.2, GraphPad, Boston, MA, USA).

Negative control, *Tp53* siRNA, *Klhl4* siRNA, F-box only protein 3 (*Fbxo3*) siRNA, and *Klhl34* siRNA were purchased from RiboBio (Guangzhou, China). Full-length cDNA of *Tp53* was amplified using PCR before being cloned into pSin-3HA vectors. Full-length cDNA of *Slc7a11* was amplified using PCR and cloned into pSin-3Flag vectors. Full-length cDNA of *Klhl4* was amplified using PCR and cloned into pSin-3HA vectors. Ubiquitin plasmids were purchased from Addgene (Cambridge, MA, USA, Cat# 12647). The mutant plasmids of *Klhl4*-Δ430-717 (Kelch domain deletion) were constructed using an overlap extension PCR method (Gu et al., 2021) and cloned into pSin-3HA vectors. The siRNA and primer sequences are listed in **Additional Table 4**. Both PC12 and HEK293 cells were transfected with siRNA or plasmids using Lipofectamine 3000 (Thermo Fisher Scientific, Cat# L3000001) according to the manufacturer’s protocols.

**Additional Table 4:** siRNA sequences and primer sequences for the plasmids

Gene	Sequence	Type
*Tp53*	5’ -GGCTCCGACTATACCACTA-3’	siRNA
*Klhl4*	5’ -GAAAGAGUUUGAUGUGAAACA-3’	siRNA
*Fbxo3*	5’ -CAUGGUUGUUAAUAGUUUAUA-3’	siRNA
*Klhl34*	5’-AGUCUAGUAUUACCGAGAAGA-3’	siRNA
*Tp53*	Forward: 5’ -CTCAAGGAGTGCGGCTAGCAATGGAGGATTCACAGTCGGATA- 3’ Reverse: 5’-CTCGAGCTCAAGCTTCGAATTCTCAGTCTGAGTCAGGCCCCA-3’	O/E
*Slc7a11*	Forward: 5’-TTGATCCTTCGAGCTAGCGGAATGGTCAGAAAGCCAGTTGTGG-3’ Reverse: 5’ -TCATGGTCTTTGTAGTCACTAGTGCAAAGCAATTTTCCTGTACTGA-3’	O/E
*Klhl4*	Forward: 5’-CTCAAGGAGTGCGGCTAGCAATGTCAGTGTCCGGCAAGAAAGAGT-3’ Reverse: 5’ -CGAGCTCAAGCTTCGAATTCTCAGTGTAGCTTCACAACAACAA-3’	
*KM4-Δ430-717*	Forward: 5’- GTGGGGGCATGAGAATTCGAAGCTTGAGCTC-3’ Reverse: 5’-TTCGAATTCTCATGCCCCCACAGTTGATTTTCTA-3’	Mutant

O/E: Overexpression; siRNA: Small interfering RNA.

### Reactive oxygen speciesdetermination

Cellular ROS were determined using flow cytometry (FCM) (*n* = 3). Briefly, PC12 cells were incubated with 10 μM DCFH-DA (S0033, Beyotime) at 37°C for 30 minutes. Subsequently, the cells were washed three times with PBS and analyzed using a flow cytometer (BD FACSVerse, BD, Franklin, NJ, USA).

### C11-BODIPY (581/591) staining

PC12 cells were cultured in medium supplemented with 10 μM C11-BODIPY (581/591) probe (RM02821, ABclonal, Wuhan, China) (*n* = 3). After 30 minutes of incubation, the medium was replaced with fresh culture medium (RPMI-1640, Gibco) devoid of the C11-BODIPY probe. Subsequently, the cells were imaged using an automated positive fluorescence microscope (DM6B, Leica). Quantitative analysis of C11-BODIPY staining was performed using ImageJ software (version 1.53c) by comparing the ratio of oxidized to non-oxidized lipids.

### Glutathione determination

A cellular GSH assay kit (S0053, Beyotime) was used to detect reduced GSH content (*n* = 3). Briefly, PC12 cells were washed with PBS, and samples were then assayed for reduced GSH according to the manufacturer’s instructions. Absorbance was measured at 412 nm using a microplate reader (BioTek, Winooski, VT, USA).

### Determination of reactive oxygen species

Cellular ROS were quantified using flow cytometry (*n* = 3). Briefly, PC12 cells were incubated with 10 μM 2′,7′-dichlorodihydrofluorescein diacetate (Beyotime, S0033) at 37°C for 30 minutes. Subsequently, the cells were washed three times with PBS, and were then analyzed using a BD FACSVerse flow cytometer (BD Biosciences, Franklin Lakes, NJ, USA).

### Intracellular ferric ion content assay

An intracellular ferric ion colorimetric assay kit (Applygen, Beijing, China; E1042) was used for the total iron content assay (*n* = 3). Briefly, PC12 cells were washed with PBS, and samples were then assayed for intracellular ferric ions according to the manufacturer’s instructions. Absorbance was measured at 550 nm using a microplate reader (BioTek).

### Chromatin immunoprecipitation-polymerase chian reaction

PC12 cells were cross-linked with 1% formaldehyde for 10 minutes at 25°C and neutralized for 5 minutes by adding glycine (to a final concentration of 0.125 M). After washing twice with cold PBS, cells were harvested and suspended in cold lysis buffer (10 mM HEPES, 85 mM KCl, 0.5% NP-40, and 0.5% PMSF). After incubation on ice for 15 minutes, cell nuclei were harvested by centrifugation at 1500 × *g* for 5 minutes. Precipitates were suspended in nuclear lysis buffer (50 mM Tris-HCl pH 7.6, 10 mM ethylenediaminetetraacetic acid [EDTA], 1% SDS, and 0.5% PMSF) and incubated for 15 minutes on ice. Subsequently, samples were sonicated on ice to achieve DNA fragments of 200–1000 bp. The ultrasound parameters were as follows: 40% amplitude, 15 cycles, cycle: pulse 7 seconds, pause 15 seconds. TP53 antibody or immunoglobulin (Ig)G control was added to the lysate and incubated overnight. Magnetic beads (L-1008 protein A/G, Bio-LinkedIn, Shanghai, China) were blocked using bovine serum albumin and single-stranded DNA for 30 minutes with rotation at 4°C, and then washed three times for 5 minutes each with dilution buffer (0.01% SDS, 1% Triton X-100, 1.2 mM EDTA, 16.7 mM Tris-HCl pH 8.1, and 167 mM NaCl). Subsequently, magnetic beads were added to the lysate for 4 hours with rotation at 4°C to precipitate the protein–DNA complex. The beads–protein–DNA complex was washed in the following order: low-salt immune complex wash buffer (0.1% SDS, 1% Triton X-100, 1 mM EDTA, 20 mM Tris-HCl pH 8.1, and 150 mM NaCl), high-salt immune complex wash buffer (0.1% SDS, 1% Triton X-100, 1 mM EDTA, 20 mM Tris-HCl pH 8.1, and 500 mM NaCl), LiCl immune complex wash buffer (250 mM LiCl, 1% NP-40, 1 mM EDTA, 10 mM Tris-HCl pH 8.1, and 1% deoxycholic acid), and Tris-EDTA buffer (5 minutes each, rotation at 4°C). The protein–DNA complex was then eluted from the magnetic beads using elution buffer (1% SDS and 100 μM NaHCO_3_, with rotation for 15 minutes at 25°C). After reverse cross-linking for 4 hours at 65°C, DNA was extracted and analyzed using PCR followed by 1.5% agarose (Sangon Biotech, Shanghai, China) gel electrophoresis. Primer sequences are listed in **Additional Table 5**.

**Additional Table 5:** Primers used for polymerase chain reaction

Gene	Sequence
*Slc7a11*	Forward: 5ʹ-CCAGCATTATTCTCCTTCA-3ʹ Reverse: 5ʹ-ACCACTTGGGTTTCTTGTC-3ʹ
*Klhl4*	Forward: 5ʹ-CGTTCTCGAAACCATGCGGA-3ʹ Reverse: 5ʹ-ACTGATTAAAATTGGGCCATGGA-3ʹ

### Cleavage under targets and tagmentation and data processing

The cleavage under targets and tagmentation (CUT&Tag) assay was performed using a Hyperactive Universal CUT&Tag Assay Kit for Illumina (Vazyme, Nanjing, China, TD903). PC12 cells were collected and washed once with wash buffer before they were bound to ConA beads (supplied with the kit) for 10 minutes at 25°C. Next, PC12 cells were resuspended in pre-cooled antibody buffer, and were then incubated with 1 μg TP53 antibody (Cell Signaling Technology, Beverly, MA, USA; Cat# 2524, RRID: AB_331743) at 4°C overnight. The next day, anti-mouse IgG (supplied with the kit) was added and incubated with gentle rotation for 1 hour at 25°C, followed by three washes with Dig-wash buffer. After incubation with 0.04 μM pA/G–Tnp for 1 hour at 25°C, samples were washed three times with Dig-300 buffer, and were then resuspended in Trueprep Tagment Buffer L (diluted in Dig-300 buffer) and incubated with gentle rotation at 37°C for 1 hour. Tagmentation was stopped by incubation with the mixed buffer, containing proteinase K, buffer L/B, and DNA extract beads, at 55°C for 10 minutes. Beads were then collected using a magnetic rack before being gently rinsed twice with 80% ethanol. DNA was eluted with ultrapure water. Libraries were established using “TD903 Hyperactive Universal CUT&Tag Assay Kit for Illumina” and “TD202 TruePrep Index Kit V2 for Illumina” (Vazyme). High-throughput sequencing was performed by the Wuhan Biobank Co., Ltd. using a Novaseq6000 (Illumina). Using a Linux operating system (Ubuntu, Canonical, London, UK), the raw sequencing data underwent quality assessment with FastQC (www.bioinformatics.babraham.ac.uk/projects/fastqc/). Post-quality control, data were mapped to the genome using Bowtie2 (http://bowtie-bio.sourceforge.net/bowtie2). Mapped data were then subjected to peak calling using Macs2. The identified peaks were annotated to corresponding genes and sorted using fold_enrichment. Thereafter, the transcription start site peak plot was visualized using the ChIPseeker package (https://bioconductor.org/packages/ChIPseeker/) within R software (version 4.2.2).

### Molecular docking prediction

The PDB files of proteins were generated using the AlphaFold Protein Structure Database (https://alphafold.ebi.ac.uk/) before being uploaded to the Global RAnge Molecular Matching Platform (GRAMM; https://gramm.compbio.ku.edu/) (Katchalski-Katzir et al., 1992). The GRAMM docking program was run to identify docking sites and calculate the binding energy. The docking model was then visualized using PyMOL software (version 2.5.5, Schrödinger LLC, New York, NY, USA).

### Coimmunoprecipitation

To investigate the effects of KLHL4 on SLC7A11 ubiquitination, HA-*Klhl4*, Flag-*Slc7a11*, and ubiquitin plasmids (Addgene, Cat# 12647) were transfected into HEK293 cells with or without siRNA-*Klhl4* transfection. To reveal the regulatory effects of the Kelch domain in KLHL4-mediated SLC7A11 ubiquitination, HA-Klhl4 (or HA-*Klhl4*-ΔKelch), Flag-*Slc7a11*, and ubiquitin plasmids were transfected into HEK293 cells. Plasmids were obtained as described in previous sections. Cells were harvested before being lysed with RIPA (with 1% PMSF) buffer. After centrifugation at 4°C (12,000 × *g* for 15 minutes), anti-Flag monoclonal antibody-conjugated beads (Selleck, Shanghai, China; B26101) were added to the lysate and rotated overnight at 4°C. For the IgG control, IgG and magnetic beads (L-1008 protein A/G) were added to the lysate with rotation overnight at 4°C. The beads were then collected and washed three times with PBS for 5 minutes each. Before undergoing western blot (WB) assay, the beads were mixed with an equal volume of 1× loading buffer and incubated in a metal bath at 95°C for 10 minutes. After transferring and blocking, the membrane was incubated overnight at 4°C with primary antibodies against HA tag (anti-HA, rabbit, 1:1000, Proteintech, Wuhan, China; Cat# 51064-2-AP, RRID: AB_11042321), Flag tag (anti-Flag; mouse, 1:5000, Sigma-Aldrich/Merck; Cat# F1804, RRID: AB_262044), and ubiquitin (anti-ubiquitin; rabbit, 1:1000, Abmart, Shanghai, China; Cat# T55965S, RRID: AB_2936388). The next day, the membrane was incubated with universal secondary antibody (light/heavy chains avoided, 1:2000, Abmart; Cat# M21008).

### Statistical analysis

Quantitative imaging data were obtained using ImageJ software (version 1.53c). Statistical analysis and data visualization were conducted using GraphPad Prism software (version 8.0.2). Data are presented as the mean ± standard deviation. Statistical analysis between two groups was performed using the unpaired *t*-test. The significance of differences between multiple groups was assessed using one-way analysis of variance (ANOVA) followed by Tukey’s multiple comparisons test. Differences in stride distance and BBB scores among the groups were evaluated via two-way ANOVA with Tukey’s *post hoc* analysis. A *P*-value < 0.05 was considered to indicate significance.

## Results

### *Tp53* is a core gene related to ferroptosis and injury severity in spinal cord injury

**Figure 1:**
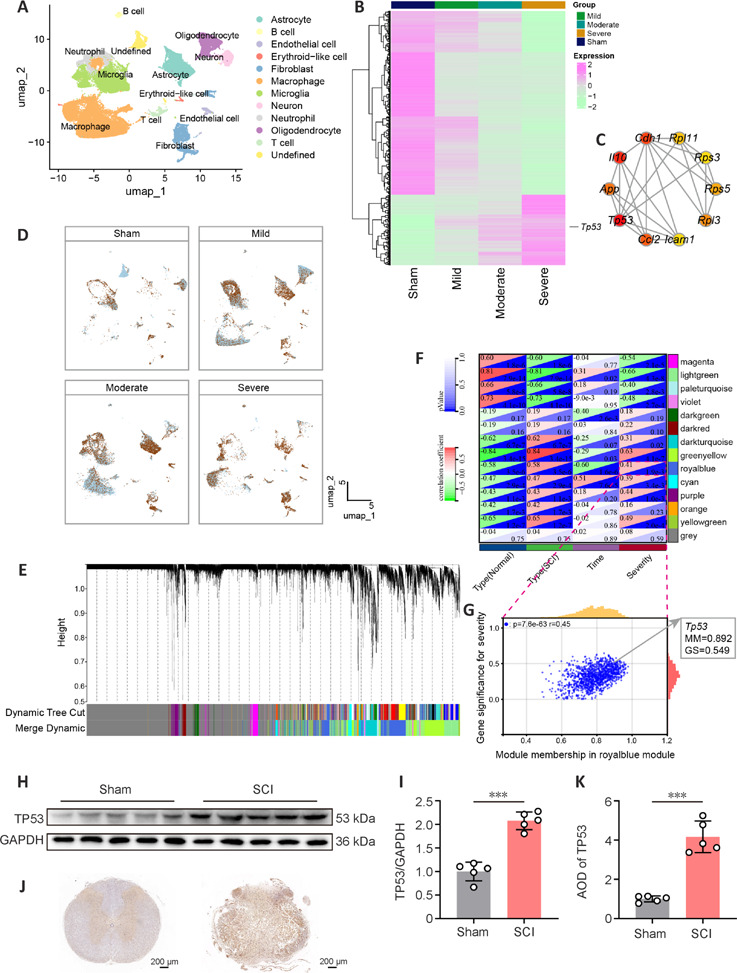
TP53 is upregulated following SCI and related to injury severity. (A) UMAP plot of rat spinal cord cells from GSE213240. (B) Heatmap of gene expression gradients in response to increasing injury severity. (C) *Tp53* was identified as the hub gene showing gradient expression changes by PPI network analyis. (D) *Tp53* expression was mapped across UMAP plots for various groups. (E) WGCNA identified co-expressed gene modules from bulk RNA-seq across three SCI models. (F) Heatmap of correlations between modules and phenotypes. (G) Scatter plot of the correlation between GS and MM in the royal blue module. (H) Western blot bands and quantification (I) of TP53 at 14 dpi (*n* = 5, unpaired *t*-test). (J) Immunohistochemical staining and quantification (K) of TP53 at 14 dpi (*n* = 5, unpaired *t*-test). The values are presented as the mean ± SD. ***P < 0.001. AOD: Average optical density; dpi: days postinjury; GS: gene significance; MM: module membership; PPI: protein‒protein interaction; SCI: spinal cord injury; WGCNA: weighted gene correlation network analysis.

In the present study, 11 cell populations were identified from the scRNA-seq data of rat spinal cords: astrocytes, B cells, endothelial cells, erythroid-like cells, fibroblasts, macrophages, microglia, neurons, neutrophils, oligodendrocytes, and T cells (**Figure 1A**). We then investigated genes that exhibited a gradient expression across varying injury severities: 451 genes were progressively upregulated and 1176 genes were progressively downregulated as injury severity increased (**Figure 1B**). *Tp53* was identified as a hub gene in this gradient shift using protein–protein interaction analysis (**Figure 1C**). Uniform manifold approximation and projection plots showed the expression of *Tp53* across various cellular populations (**Figure 1D**). WGCNA was performed following the normalization of three datasets (**Additional Figure 1A** and **B**). In total, 14 gene modules were identified; modules darkturquoise, greenyellow, royalblue, cyan, purple, and yellowgreen showed significant positive correlations with injury severity (**Figure 1E** and **F**). Within the royalblue module, *Tp53* emerged as one of the leading genes (**Figure 1G**). Gene set enrichment analysis indicated that ferroptosis was significantly upregulated across three distinct SCI models, and *Tp53* was included in the leading edge subset within 60 dpi (**Additional Figure 1C**). Finally, through differential gene expression analysis and animal experiments (WB and immunohistochemistry) at 14 dpi, we revealed the aberrant upregulation of TP53 after SCI (**Figure 1H–K** and **Additional Figure 1D**).

### Inhibition of TP53 accelerates neural function recovery in rats with spinal cord injury

**Figure 2:**
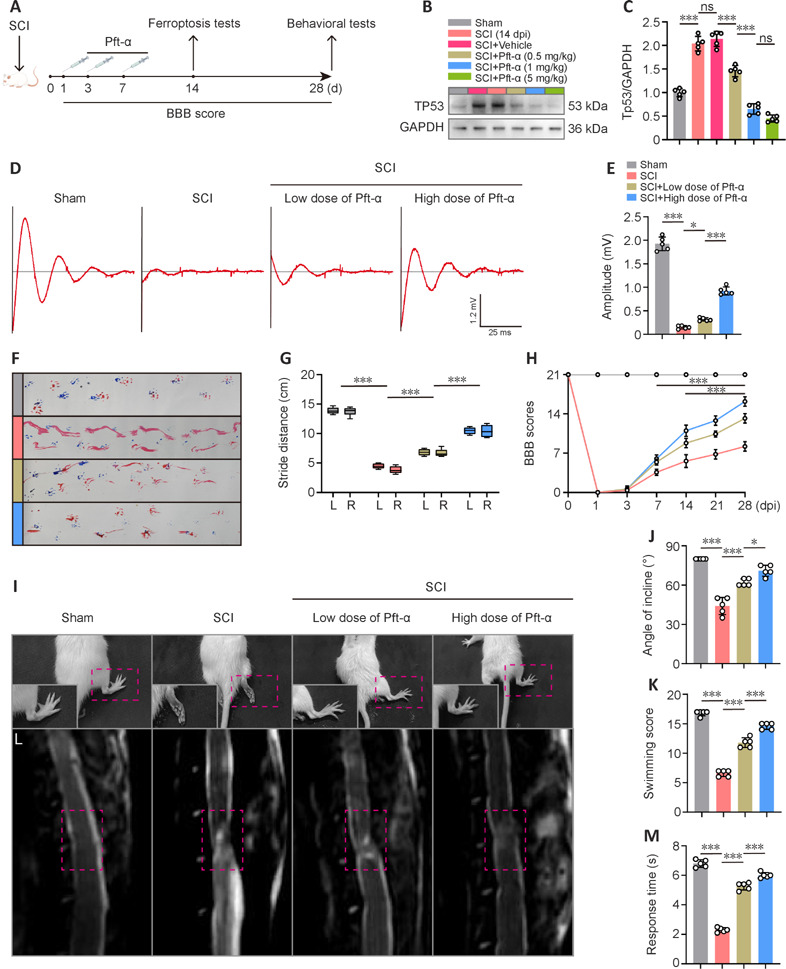
Inhibition of TP53 accelerates neural function recovery. (A) Schematic of the animal experiments. (B, C) Western blot bands (B) and quantification (C) of the inhibitory effects of different concentrations of Pft-α on TP53 expression after SCI (*n* = 5, one-way ANOVA followed by Tukey’s multiple comparison test). (D) Representative images of electrophysiological traces with quantitative statistics of the amplitude (E) of MEPs at 28 dpi (*n* = 5, one-way ANOVA followed by Tukey’s multiple comparison test). (F) Representative footprints with quantitative statistics (G) of the stride distance at 28 dpi (*n* = 5, two-way ANOVA followed by Tukey’s multiple comparison test). (H) BBB scores of the rats in the four groups (*n* = 5; two-way ANOVA followed by Tukey’s multiple comparison test). (I) Representative images of oblique board tests at 28 DPI. The boxed border regions are enlarged. (J) Quantitative statistics of the oblique board test (*n* = 5, one-way ANOVA followed by Tukey’s multiple comparison test). (K) LSS scores of the rats in the four groups at 28 DPI (*n* = 5; one-way ANOVA followed by Tukey’s multiple comparison test). (L) Representative individual MR images at 28 DPI. The epicenter is highlighted by a boxed border. (M) Response time of rats in the photothermal sensitivity test at 28 DPI (*n* = 5; one-way ANOVA followed by Tukey’s multiple comparison test). The values are presented as the mean ± SD. *P < 0.05, ***P < 0.001. ANOVA: Analysis of variance; BBB: Basso, Beattie, and Bresnahan scale; dpi: days post-injury; L: left; MEP: Motor evoked potential; MRI: Magnetic resonance imaging; ns: not significant; Pft-α: pifithrin-α (TP53 inhibitor); R: right; SCI: spinal cord injury; TP53: tumor protein 53.

We inhibited TP53 in rats with SCI via the intraperitoneal injection of PFT-α (**Figure 2A**). The inhibitory effect of PFT-α on TP53 was validated using WB analysis (**Figure 2B** and **C**). The 1 mg/kg concentration of PFT-α was more effective than the 0.5 mg/kg concentration (*P* < 0.001), but there was no significant difference between 5 mg/kg and 1 mg/kg (*P* = 0.134). Thus, PFT-α at 0.5 mg/kg (low dose) and 1 mg/kg (high dose) were selected to investigate the effects of TP53 inhibition on neural function recovery after SCI.

Electrophysiological analysis was conducted to further evaluate functional recovery using MEPs, which are a recognized indicator of electrical signal conduction capacity (Lin et al., 2022a). In the SCI group, only a weak signal above background levels was detected. Both low- and high-dose PFT-α significantly enhanced MEP amplitude compared with the SCI group (**Figure 2D** and **E**). The footprint analysis, BBB scale, oblique board test, and swimming test were performed to assess motor function recovery, and the plantar photothermal sensitivity test was conducted to assess sensory function recovery (Tan et al., 2024). Footprints of the SCI group showed distinct indications of dragging, and no paw prints were recorded. Paw prints in the PFT-α groups indicated that the rats exhibited persistent sole-bearing movements (**Figure 2F**). In addition, the stride distances of rats in low- and high-dose PFT-α groups were significantly larger than those in the SCI group (**Figure 2G**). In terms of BBB scores, there were no significant differences between the SCI and PFT-α groups within 3 dpi. However, from 7 dpi, the BBB scores of rats in the low- and high-dose PFT-α groups were significantly higher than those of rats in the SCI group (all *P* < 0.001; **Figure 2H**). In the oblique board and swimming tests, rats in the PFT-α groups exhibited superior performance compared with the SCI group (**Figure 2I–K**). We further evaluated injured spinal cord recovery using magnetic resonance imaging, which revealed that the hyperintense region of the lesion was narrower in PFT-α groups than in the SCI group (**Figure 2L**). After SCI, there is reportedly a reduction in the thermal nociception threshold, resulting in hyperalgesia (McFarlane et al., 2020; Wan et al., 2020; Zhang et al., 2023b). In the present study, treatment with PFT-α significantly prolonged the response time relative to the SCI group (low-dose PFT-α *vs*. SCI: *P* < 0.001; high-dose PFT-α *vs*. SCI: *P* < 0.001), indicating improvements in thermal hyperalgesia after SCI (**Figure 2M**). In the aforementioned tests, rats treated with high-dose PFT-α exhibited significantly superior performance compared with the low-dose group. High-dose PFT-α (1 mg/kg) was therefore selected for subsequent tests.

### Inhibition of TP53 alleviates ferroptosis after spinal cord injury

**Figure 3:**
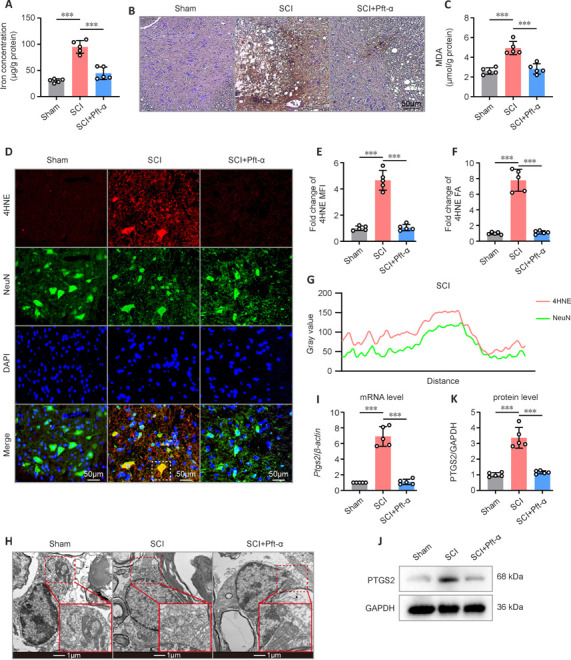
Inhibition of TP53 mitigates SCI-induced ferroptosis. (A) Tissue iron content in the spinal cords of the three groups at 14 dpi (*n* = 5; one-way ANOVA followed by Tukey’s multiple comparison test). (B) Prussian blue staining (enhanced with 3,3′-diaminobenzidine) of spinal cord sections from the three groups at 14 dpi. (C) MDA detection in injured spinal cords at 14 dpi (*n* = 5; one-way ANOVA followed by Tukey’s multiple comparison test). (D) Immunofluorescence images of 4HNE (red) and NeuN (green) cells at 14 dpi. (E, F) Quantification of 4HNE immunofluorescence (*n* = 5; one-way ANOVA followed by Tukey’s multiple comparison test). (G) Colocalization analysis of 4HNE and NeuN in the dashed box of the SCI group. (H) Transmission electron microscopy images of mitochondria in the spinal cords of the three groups at 14 dpi. (I) *Ptgs2* mRNA levels in the three groups were detected via qPCR at 14 dpi (*n* = 5, one-way ANOVA followed by Tukey’s multiple comparison test). (J, K) Western blot bands (J) and quantification (K) of PTGS2 expression in the three groups at 14 dpi (*n* = 5, one-way ANOVA followed by Tukey’s multiple comparison test). The values are presented as the mean ± SD. ***P < 0.001. 4HNE: 4-Hydroxynonenal; ANOVA: analysis of variance; DAPI: 4′,6-diamidino-2-phenylindole; dpi: days post-injury; FA: fluorescence area; MDA: malondialdehyde; MFI: mean fluorescence intensity; NeuN: neuronal nuclei; ns: not significant; Pft-α: pifithrin-α (TP53 inhibitor); PTGS2: prostaglandin-endoperoxide synthase 2; SCI: spinal cord injury.

The characteristic manifestations of ferroptosis after SCI include iron overload, the accumulation of lipid peroxides, and mitochondrial dysfunction (Dixon et al., 2012). There was significant iron overload within the lesion epicenter at 14 dpi, characterized by increased local iron content and enlarged areas positive for Prussian blue and FerroOrange staining. By contrast, rats treated with PFT-α did not exhibit significant indicators of iron overload (**Figure 3A** and **B** and **Additional Figure 2A** and **B**).

**Figure 4:**
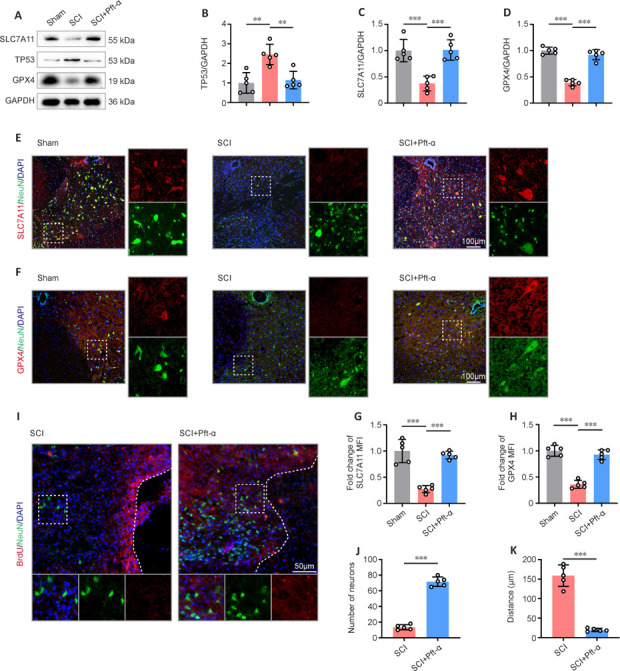
Inhibition of TP53 promotes neuronal survival by activating SLC7A11 and GPX4 after SCI. (A–D) Western blot bands (A) and quantification (B, C, D) of TP53, SLC7A11 and GPX4 at 14 dpi (*n* = 5; one-way ANOVA followed by Tukey’s multiple comparison test). (E) Immunofluorescence images of SLC7A11 and NeuN at 14 dpi. (F) Immunofluorescence images of GPX4 and NeuN at 14 dpi. (G) Quantitative statistics of SLC7A11 immunofluorescence (*n* = 5, one-way ANOVA followed by Tukey’s multiple comparison test). (H) Quantitative statistics of GPX4 immunofluorescence (*n* = 5; one-way ANOVA followed by Tukey’s multiple comparison test). (I) Immunofluorescence images of BrdU and NeuN staining at 14 dpi. (J) Quantitative statistics for the number of neurons (*n* = 5, unpaired *t*-test). (K) Quantitative statistics of the distances from neurons to the injury boundary (*n* = 5, unpaired *t*-test). The boxed border regions are enlarged. The values are plotted as the mean ± SD. ***P < 0.001. ANOVA: Analysis of variance; BrdU: 5-bromo-2′-deoxyuridine; DAPI: 4′,6-diamidino-2-phenylindole; dpi: days post-injury; FA: fluorescence area; GAPDH: glyceraldehyde 3-phosphate dehydrogenase; GPX4: glutathione peroxidase 4; MFI: mean fluorescence intensity; NeuN: neuronal nuclei; Pft-α: pifithrin-α (TP53 inhibitor); SCI: spinal cord injury; TP53: tumor protein 53.

MDA and 4-hydroxynonenal (4HNE) are terminal products of lipid peroxidation (Xu et al., 2020). Liperfluo is a fluorescent probe that is specifically used to label peroxidized lipids because of its high selectivity for peroxidized phosphatidylcholine (Liu et al., 2023b). After SCI, we observed a notable increase in lipid peroxides (marked by Liperfluo, **Additional Figure 2C** and **D**) and their products, MDA (**Figure 3C**) and 4HNE (**Figure 3D–F**), within the lesion epicenter. Intriguingly, the IF results suggested that 4HNE may co-localize with the neuronal biomarker NEUN in the SCI group (**Figure 3G**), indicating a significant accumulation of lipid peroxides within neurons post-SCI. However, the aberrant accumulation of lipid peroxidation was largely mitigated by PFT-α administration. Additionally, PFT-α significantly reduced the cavity area (**Additional Figure 3A** and **B**) and increased the number of Nissl^+^ neurons within the lesion epicenter after SCI (**Additional Figure 3C** and **D**).

During ferroptosis, mitochondria exhibit morphological alterations, including shrinkage and the disruption or reduction of cristae (Yuan et al., 2022). In the present study, we observed that mitochondria were notably shrunken and cristae were disrupted in the SCI group compared with the Sham group, whereas the morphology of mitochondria in the PFT-α group more closely resembled the normal state (**Figure 3H**). Additionally, the expression of prostaglandin-endoperoxide synthase 2 (PTGS2), a recognized ferroptosis indicator, was significantly downregulated in the PFT-α group compared with the SCI group (qRT-PCR: PFT-α *vs.* SCI, *P* < 0.001; WB: PFT-α *vs.* SCI, *P* < 0.001; **Figure 3I–K**). Overall, our findings suggest that TP53 inhibition may mitigate SCI-induced ferroptosis.

### Inhibition of TP53 enhances neuronal survival by activating SLC7A11 and glutathione peroxidase 4

SLC7A11 is regulated by TP53 and affects the enzymatic activity of glutathione peroxidase 4 (GPX4) (Jiang et al., 2015; Liu et al., 2023a). We therefore investigated the expression levels of SLC7A11 and GPX4 with PFT-α treatment post-SCI. Inhibition of TP53 resulted in the marked restoration of SLC7A11 and GPX4 levels (**Figure 4A–D**). Additionally, IF results indicated that SLC7A11 and GPX4 were significantly diminished in the SCI group, and TP53 inhibition increased SLC7A11 and GPX4 immunoreactivities within neurons (**Figure 4E–H**). To further explore the effects of TP53 inhibition on neurons, we labeled newborn neurons using BrdU (a recognized biomarker for proliferating cells) and NeuN (**Figure 4I**). No clear co-localization between BrdU and NeuN was observed in either the SCI or PFT-α groups. Notably, the PFT-α group exhibited significantly more neurons than the SCI group (*P* < 0.001), and these neurons were situated closer to the injury boundary (*P* < 0.001; **Figure 4J** and **K**).

### Inhibition of TP53 facilitates the re-establishment of neural circuits

**Figure 5:**
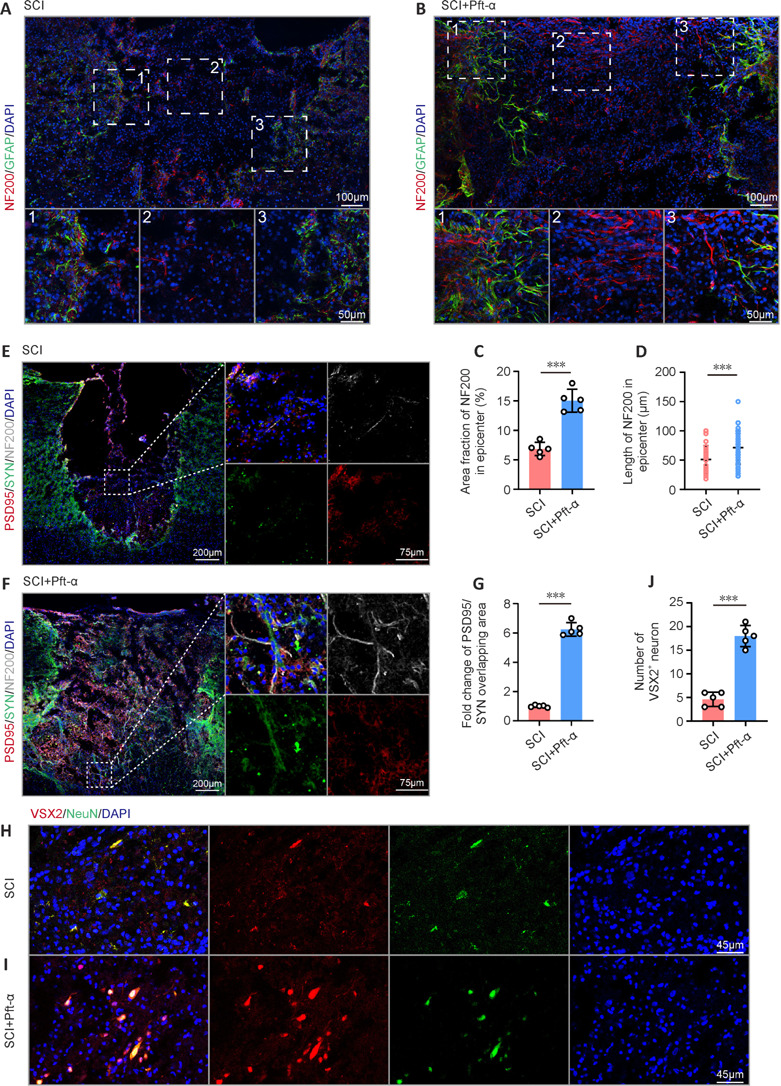
Inhibition of tumor protein 53 prompts neural circuit reestablishment in spinal cord tissue of rats. (A, B) Immunofluorescence images of NF200 and GFAP at 14 dpi. (C, D) Quantitative statistics for the positive area and length of NF200 (*n* = 5, unpaired *t*-test). (E, F) Immunofluorescence images of NF200, SYN and PSD95 at 14 dpi. (G) Quantitative statistics for the overlapping area of SYN and PSD95 (*n* = 5, unpaired *t*-test). (H, I) Immunofluorescence images of VSX2 and NeuN at 14 dpi. (J) Quantitative statistics for the number of VSX2^+^ neurons (*n* = 5, unpaired *t*-test). The boxed border regions are enlarged. The values are presented as the mean ± SD. ***P < 0.001. DAPI: 4′,6-Diamidino-2-phenylindole; dpi: days post-injury; GFAP: glial fibrillary acidic protein; NeuN: neuronal nuclei; NF200: neurofilament 200; Pft-α: pifithrin-α (TP53 inhibitor); PSD95: postsynaptic density protein 95; SCI: spinal cord injury; SYN: synaptophysin; VSX2: visual system homeobox 2.

The characterization of neurofilament 200 (NF200) is a pivotal methodology in SCI-related research, offering essential insights into axonal damage, repair processes, and regenerative mechanisms (Jing et al., 2021; Tan et al., 2024). Glial fibrillary acidic protein (GFAP) is a well-known biomarker for glial scars, and is frequently used to delineate injury boundaries in SCI-related research (Cooper et al., 2020; Tan et al., 2024). In the SCI group, the lesion epicenter was encapsulated by a glial scar (marked by GFAP) that delineated a boundary with the adjacent normal tissue. Moreover, a few NF200^+^ axon fragments were observed within the epicenter. In the PFT-α group, a significant increase in NF200^+^ fibers was observed within the lesion epicenter. Statistical analysis revealed that the NF200^+^ area and fiber length in the epicenter of the PFT-α group were significantly greater than those in the SCI group (area fraction: *P* < 0.001; length: *P* = 0.001; **Figure 5A–D**).

Synaptophysin (SYN) and postsynaptic density protein 95 (PSD95) are well-recognized biomarkers of presynaptic and postsynaptic membranes (Zhang et al., 2017; Li et al., 2017). In the PFT-α group, SYN and PSD95 signals were significantly enhanced relative to the SCI group. Furthermore, the overlapping area of SYN/PSD95 was significantly increased by treatment with PFT-α (*P* < 0.001; **Figure 5E–G**).

Two recent studies identified V2a neurons as a cluster of neurons that upregulate neural circuit remodeling programs in SCI (Squair et al., 2023; Skinnider et al., 2024). V2a neurons in the present study were co-labeled using visual system homeobox 2 (VSX2) and NeuN. The number of V2a neurons was significantly increased after treatment with PFT-α (*P* < 0.001; **Figure 5H–J**).

### Knockdown of *Tp53* strengthens the anti-ferroptotic capacity of PC12 cells

**Figure 6:**
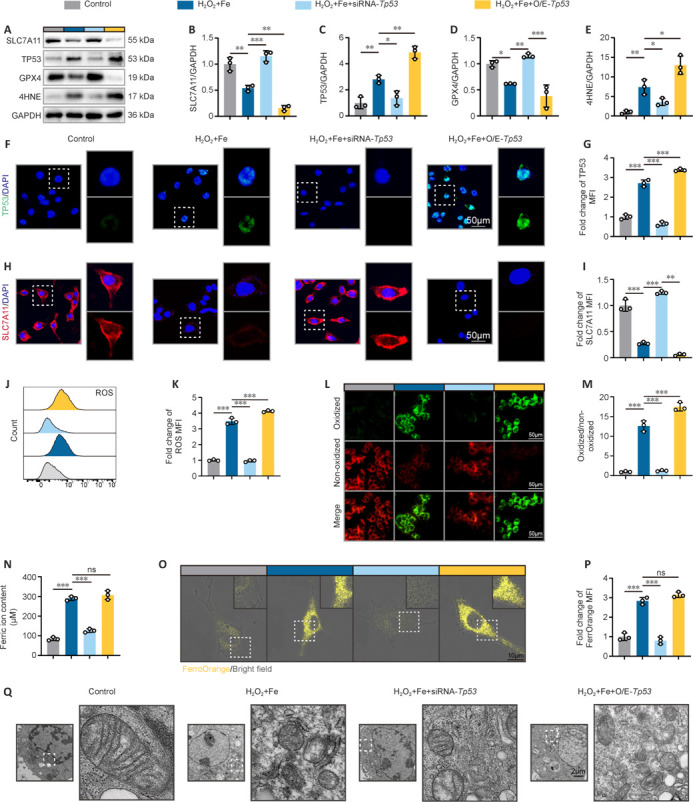
TP53 manipulates ferroptosis induced by H_2_O_2_ and Fe in PC12 cells. (A–E) Western blot bands (A) and quantification (B–E) of SLC7A11, TP53, GPX4 and 4HNE (*n* = 3, ANOVA followed by Tukey’s multiple comparison test). (F) Immunofluorescence images and quantitative statistics (G) of TP53 in PC12 cells (*n* = 3; one-way ANOVA followed by Tukey’s multiple comparison test). (H) Immunofluorescence images and quantitative statistics (I) of SLC7A11 in PC12 cells (*n* = 3, one-way ANOVA followed by Tukey’s multiple comparison test). (J) Flow cytometry detection and quantification (K) of ROS (*n* = 3, one-way ANOVA followed by Tukey’s multiple comparison test). (L) Fluorescence images and quantification (M) of C11-BODIPY (581/591) (*n* = 3, one-way ANOVA followed by Tukey’s multiple comparison test). (N) Intracellular ferric ion content determination (*n* = 3; one-way ANOVA followed by Tukey’s multiple comparison test). (O) Fluorescence images and quantitation (P) of FerroOrange (*n* = 3, one-way ANOVA followed by Tukey’s multiple comparison test). (Q) Transmission electron microscopy images of mitochondria in PC12 cells. The boxed border regions are enlarged. The values are plotted as the mean ± SD. *P < 0.05, **P < 0.01, ***P < 0.001. 4HNE: 4-Hydroxynonenal; ANOVA: analysis of variance; DAPI: 4′,6-diamidino-2-phenylindole; GAPDH: glyceraldehyde 3-phosphate dehydrogenase; GPX4: glutathione peroxidase 4; MFI: mean fluorescence intensity; ns: not significant; O/E: overexpression; ROS: reactive oxygen species; TP53: tumor protein 53.

To further investigate the regulatory mechanisms of TP53 on neuronal ferroptosis, PC12 cells were used to establish a cell model of SCI-induced ferroptosis by treatment with H_2_O_2_ and Fe. The cell model was then validated using a ferroptosis inducer (erastin) and inhibitor (ferrostatin-1) (**Additional Figure 4**). Subsequently, PC12 cells were transfected with *Tp53* siRNA and *Tp53* O/E plasmid. Transfection efficiency was confirmed using green fluorescent protein and WB assays (**Additional Figure 5A–E**). The WB and IF assays revealed that *Tp53* siRNA notably elevated the protein levels of SLC7A11 and GPX4 and decreased the content of 4HNE in our cell model (**Figure 6A–I** and **Additional Figure 5F–I**). Moreover, within the H_2_O_2_ + Fe + siRNA-*Tp53* group, there was a significant increase in intracellular GSH content (**Additional Figure 5J**). ROS, lipid ROS, and MDA were significantly reduced by *Tp53* knockdown relative to the H_2_O_2_ + Fe group (ROS: *P* < 0.001; lipid ROS: *P* < 0.001; and MDA: *P* < 0.001; **Figure 6J–M** and **Additional Figure 5K**). By contrast, *Tp53*-O/E exhibited the opposite trend. Both the intracellular ferric ion assay and FerroOrange staining were used to characterize iron content in each group. Iron overload was alleviated in the H_2_O_2_ + Fe + siRNA-*Tp53* group, but was not aggravated in the H_2_O_2_ + Fe + O/E-*Tp53* group relative to the H_2_O_2_ + Fe group (**Figure 6N–P**). TEM was used to observe mitochondrial morphology; mitochondrial cristae were clearly visible in the Control and H_2_O_2_ + Fe + siRNA-*Tp53* groups, whereas in the H_2_O_2_ + Fe and H_2_O_2_ + Fe + O/E-*Tp53* groups, cristae were disrupted or disappeared (**Figure 6Q**).

### TP53 modulates SLC7A11 expression via transcriptional and post-translational modification mechanisms

**Figure 7:**
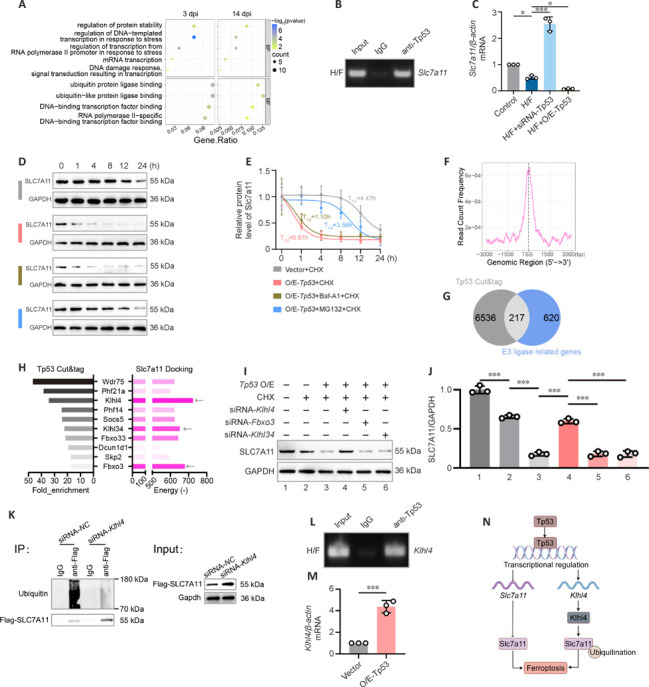
TP53 regulates the transcription and ubiquitination of SLC7A11. (A) *Tp53* was involved in the transcription-related and ubiquitination-related BP/MF terms in the GO enrichment analysis of contusion SCI. (B) ChIP‒PCR of *Slc7a11*. (C) Quantitative reverse transcription polymerase chain reaction of *Slc7a11* (*n* = 3, one-way ANOVA followed by Tukey’s multiple comparison test). (D) The protein decay assay and half-life (E) of SLC7A11. (F) TSS peak plot of TP53 CUT&Tag. (G) In total, 217 E3 ligase-related genes were regulated by Tp53. (H) Binding energy with SLC7A11 of the top 10 genes in the TP53 CUT&Tag. (I, J) Western blot bands (I) and quantification (J) were used to evaluate the effects of *Klhl4*, *Fbxo3*, and *Klhl34* knockdown on TP53-mediated SLC7A11 degradation (*n* = 3; one-way ANOVA followed by Tukey’s multiple comparison test). (K) Coimmunoprecipitation-western blot analysis of the effects of KLHL4 on SLC7A11 ubiquitination. (L) ChIP‒PCR of *Klhl4*. (M) Quantitative reverse transcription polymerase chain reaction of *Klhl4* (*n* = 3, unpaired *t*-test). (N) Schematic diagram of SLC7A11 regulation by TP53. The values are presented as the mean ± SD. *P < 0.05, ***P < 0.001. ANOVA: Analysis of variance; BP: biological process; ChIP‒PCR: chromatin immunoprecipitation-PCR; dpi: days post-injury; GAPDH: glyceraldehyde 3-phosphate dehydrogenase; H/F: H_2_O_2_+Fe; KLHL: Kelch-like protein; MF: molecular function; O/E: overexpression; TP53: tumor protein 53; TSS: transcription start site.

The Gene Ontology analysis of contusion SCI suggested that TP53 may be involved in mRNA transcription and protein ubiquitination (**Figure 7A**). TP53 negatively regulated *Slc7a11* transcription, which aligns with a previous report (Jiang et al., 2015; **Figure 7B** and **C**). To ascertain whether ubiquitination plays a role in TP53-mediated SLC7A11 exhaustion, we investigated the degradation of SLC7A11 in the presence and absence of *Tp53*-O/E. In the presence of CHX (a protein synthesis inhibitor), *Tp53*-O/E significantly accelerated the degradation of SLC7A11. This degradation process was significantly impeded by MG132 (a potent proteasome inhibitor) but not Baf-A1 (an autophagosome–lysosome pathway blocker) (**Figure 7D** and **E**). The CUT&Tag assay was used in our cell model to investigate which ubiquitination-related genes are regulated by TP53. The peak plot derived from our CUT&Tag analysis exhibited a distinct enrichment of TP53 binding near the transcription start site (**Figure 7F**). In total, 217 E3 ligase-related genes were screened out from the target gene of *Tp53* (**Figure 7G**). These genes were ranked based on fold_enrichment data derived from TP53 CUT&Tag bioinformatic analysis, and corresponding proteins of the top 10 candidates were subjected to molecular docking with SLC7A11. KLHL4, FBXO3, and KLHL34 were identified as the top three candidates with the highest potential to bind to SLC7A11 (**Figure 7H** and **Additional Figure 6**). We then examined the effects of individually knocking down each of these three genes on SLC7A11 expression. Knockdown of *Klhl4* significantly ameliorated TP53-mediated SLC7A11 depletion (**Figure 7I** and **J**) and reduced the level of SLC7A11 ubiquitination (**Figure 7K**). Moreover, we confirmed the positive regulatory effect of TP53 on *Klhl4* transcription via chromatin immunoprecipitation PCR and qRT-PCR (**Figure 7L** and **M**). In light of these findings, we propose that TP53 may play a role in the regulation of *Slc7a11* mRNA transcription and the ubiquitination of its corresponding protein (**Figure 7N**).

### Kelch-like protein 4 interacts with SLC7A11 via the Kelch domain to facilitate ubiquitination

**Figure 8:**
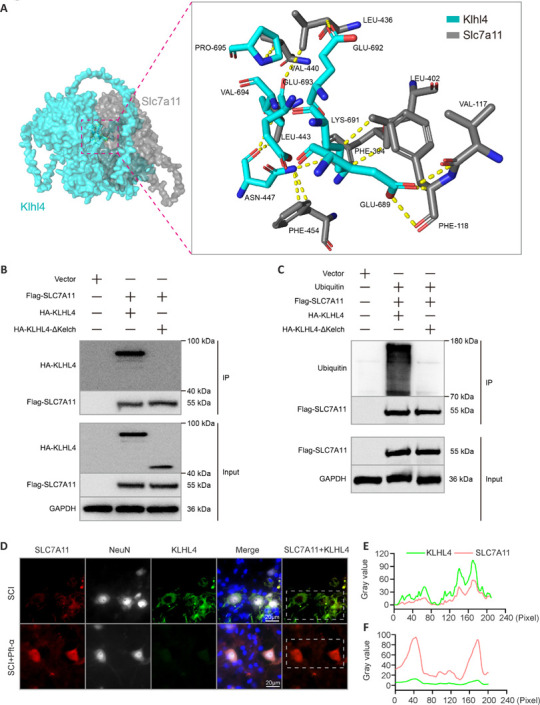
KLHL4 interacts with SCL7A11 via the Kelch domain to facilitate ubiquitination. (A) Molecular docking model of KLHL4 and SLC7A11. (B) CoIP-WB analysis of the impact of Kelch domain deletion on the interaction between KLHL4 and SLC7A11. (C) CoIP-WB was used to detect the impact of Kelch domain deletion on SLC7A11 ubiquitination. (D) Immunofluorescence images of KLHL4, SLC7A11 and NeuN expression at 14 days post-injury. (E) Co-localization analysis of KLHL4 and SLC7A11 in the dashed boxes of the SCI group. (F) Co-localization analysis of KLHL4 and SLC7A11 in the dashed boxes of the SCI + Pft-α group. CoIP-WB: Coimmunoprecipitation-western blot. KLHL: Kelch-like protein; NeuN: neuronal nuclei; Pft-α: pifithrin-α (TP53 inhibitor); SCI: spinal cord injury.

To further investigate the regulatory role of KLHL4 in SLC7A11 ubiquitination, we predicted their interaction using molecular docking analysis. Results indicated that SLC7A11 binds to KLHL4 through the Kelch domain (**Figure 8A**), consistent with the reported substrate recognition mechanisms of KLHL4 (Yumimoto et al., 2023). Subsequently, we constructed KLHL4 mutants lacking the Kelch domain (KLHL4-ΔKelch). Intriguingly, KLHL4 interacted with SLC7A11 and facilitated its ubiquitination; however, the Kelch domain deletion disrupted this molecular interaction and decreased the SLC7A11 ubiquitination level (**Figure 8B** and **C**). Finally, we validated the interaction between KLHL4 and SLC7A11 *in vivo*. IF analysis suggested that KLHL4 and SLC7A11 co-localized within neurons post-SCI, although this co-localization was largely abolished by PFT-α (**Figure 8D–F**).

## Discussion

In the present study, we predicted the important role of TP53 in SCI through a comprehensive bioinformatic analysis. After SCI, TP53 expression was upregulated and was significantly enriched in processes related to mRNA transcription and protein ubiquitination. Inhibition of TP53 markedly alleviated the ferroptotic phenotype in rats with SCI and accelerated neural function recovery. *In vitro*, we confirmed the negative transcriptional regulation exerted by TP53 on *Slc7a11*, a finding that aligns with previous studies (Jiang et al., 2015). Additionally, our research revealed that TP53 can amplify SLC7A11 ubiquitination by promoting *Klhl4* transcription. Using both *in vivo* and *in vitro* experiments, our research indicates that TP53 targets SLC7A11 to modulate SCI-induced neuronal ferroptosis.

Ferroptosis is an iron-dependent, non-apoptotic form of programmed cell death (Dixon et al., 2012). Pathological changes post-SCI, such as hemorrhage accumulation, theoretically support the occurrence of ferroptosis (Li and Jia, 2023). In the present study, we characterized iron overload in spinal cord tissue after SCI, consistent with previous reports (Kang et al., 2023). Moreover, TP53 reportedly regulates intracellular iron homeostasis, and iron overload affects the molecular function of TP53 (Zhang et al., 2008; Shen et al., 2014). Herein, we demonstrated that TP53 inhibition post-SCI significantly alleviated iron overload at both the tissue and cellular levels. Additionally, we provided *in vivo* and *in vitro* evidence that TP53 inhibition may protect neurons from SCI-induced ferroptosis by activating the SLC7A11–GPX4 pathway. On the basis of our experimental results, we hypothesize that TP53 inhibition activates the SLC7A11–GPX4 pathway, thereby restoring iron homeostasis by ameliorating the redox imbalance.

Neuronal survival is critical for neural function recovery after SCI (Zhang et al., 2023a). In the present study, TP53 inhibition alleviated the ferroptotic phenotype and improved neural function in rats with SCI. In regions adjacent to the lesion epicenter, the PFT-α-treated group displayed notably higher neuronal counts than the SCI group. Additionally, a distinct separation between NEUN and BRDU fluorescent signals indicated that TP53 inhibition may promote neuronal survival in the injured spinal cord. The enhancement of neuronal survival is a fundamental prerequisite for the re-establishment of neural circuits post-SCI

Neural circuit re-establishment constitutes a foundational element for functional recovery following SCI (Ke et al., 2023). Axonal regeneration and synaptic connections are essential for neural circuit re-establishment (Borger et al. 2022; Newman et al., 2022). V2a neurons are a critical neuronal population within the spinal cord, and play a vital role in the regulation of motor control and neural conduction (Squair et al., 2023; Skinnider et al., 2024). In the current study, the PFT-α-treated group displayed a pronounced increase in axonal signals within the epicenter, which was characterized by longer extensions and a more orderly distribution. More importantly, in the PFT-α-treated group, an increased overlap of pre- and post-synaptic membranes along axons was observed within the lesion epicenter; this was accompanied by a higher survival rate of V2a neurons. Together, these findings suggest that TP53 inhibition may foster neural circuit re-establishment, thus helping to explain the significant enhancement in neural function that was observed in rats treated with PFT-α.

Motivated by our bioinformatic analysis, we explored the regulatory effects of TP53 on SLC7A11 ubiquitination. The ubiquitin–proteasome pathway serves as the main pathway for intracellular protein degradation, requiring three classes of enzymes: E1 ubiquitin-activating enzymes (E1), E2 ubiquitin-conjugating enzymes (E2), and E3 ubiquitin ligases (E3) (Cruz Walma et al., 2022). The process initiates with the ATP-dependent activation of ubiquitin by E1. Subsequently, E1 transfers ubiquitin to E2, generating a ubiquitin–E2 complex. Eventually, the ubiquitin-E2–complex interacts with E3, which is responsible for selecting target proteins and directing the attachment of ubiquitin to these targets (Wirianto et al., 2020). We therefore focused on E3 ligase-related genes regulated by TP53, and demonstrated that TP53 promoted SLC7A11 ubiquitination by activating *Klhl4* transcription.

KLHL4 is an adaptor in the ubiquitination process; it assists E3 ligases in substrate recognition (Asmar et al., 2023). The Kelch-repeat domain of KLHL4 has been reported to mediate the recognition and binding of substrates (Yumimoto et al., 2023). In the present study, SLC7A11 was identified to bind tightly with the Kelch domain of KLHL4 in a molecular docking analysis. Deletion of the Kelch domain attenuated this interaction and reduced SLC7A11 ubiquitination. Moreover, IF staining suggested that SLC7A11 and KLHL4 were co-localized within neurons post-SCI. Collectively, our findings suggest that, by recognizing SLC7A11 as a substrate via the Kelch domain, KLHL4 is involved in regulating TP53-mediated SLC7A11 ubiquitination.

There are two major limitations in our study. First, although we characterized the co-localization of KLHL4 and SLC7A11 in neurons post-SCI, further *in vivo* confirmation of the role of KLHL4 (e.g., using conditional knockout animal models) would substantially strengthen the conclusions of our study. Second, although PFT-α is a well-established TP53 inhibitor, it exhibits inherent limitations; complementary approaches (e.g., using adeno-associated virus-based gene editing) may provide more comprehensive insights into the role of TP53 in SCI.

In conclusion, the present study sheds light on the mechanisms underlying SCI-induced neuronal ferroptosis, thus providing potential therapeutic targets. Our findings highlight the pivotal role of SLC7A11 in neuronal survival, which is critical for neural circuit re-establishment and neural function recovery after SCI. The insights from our study may contribute to the development of more effective treatments for SCI through the strategic modulation of ferroptosis.

## Additional files:

***Additional Figure 1:*** *Tp53 is identified as a core gene involved in ferroptosis across different SCI models.*

Additional Figure 1 *Tp53* is identified as a core gene involved in ferroptosis across different SCI models.
(A) UMAP plots of three SCI bulk RNA-seq data before batch correction. (B) UMAP plots of three SCI bulk RNA sequencing data after batch correction. (C) *Tp53* was the core gene involved in ferroptosis in the three SCI models. (D) *Tp53* was upregulated in the three SCI models. DPI: Days post-injury; SCI: spinal cord injury; Tp53: tumor protein 53

***Additional Figure 2:*** *Inhibition of TP53 eliminates ferric ion and lipid peroxides within the lesion epicenter.*

Additional Figure 2 Inhibition of TP53 eliminates ferric ion and lipid peroxides within the lesion epicenter.
(A) Fluorescence images and quantitation (B) of FerroOrange (*n*=5, one-way analysis of variance followed by Tukey’s multiple comparison test). (C) Fluorescence images and quantification (D) of Liperfluo (*n*=5, one-way analysis of variance followed by Tukey’s multiple comparison test). The values are presented as the mean ± SD. ***P < 0.001. DAPI: 4',6-Diamidino-2-phenylindole; FA: fluorescence area; MFI: mean fluorescence intensity; Pft-α: pifithrin-α (TP53 inhibitor).

***Additional Figure 3:*** *Inhibition of TP53 minimizes the extent of the cavity and increased the neuronal population after SCI.*

Additional Figure 3 Inhibition of TP53 minimizes the extent of the cavity and increased the neuronal population after SCI.
(A) Representative images and cavity area quantitation (B) for HE staining (*n*=5, one-way analysis of variance followed by Tukey’s multiple comparison test). (C) Representative images and quantification (D) of Nissl-stained samples (*n*=5; one-way analysis of variance followed by Tukey’s multiple comparison test). The boxed border regions are enlarged. The values are presented as the mean ± SD. ***P < 0.001. HE: Hematoxylin‒eosin; MFI: mean fluorescence intensity; Pft-α: pifithrin-α (TP53 inhibitor).

***Additional Figure 4:*** *In vitro model establishment and validation of spinal cord-induced ferroptosis.*

Additional Figure 4 *In vitro* model establishment and validation of spinal cord-induced ferroptosis.
(A) Western blot and quantification (B, C) of SLC7A11 and GPX4 in the four groups (*n*=3; one-way ANOVA followed by Tukey’s multiple comparison test). (D) Fluorescence images and quantification (E) of C11-BODIPY (581/591) (*n*=3, one-way ANOVA followed by Tukey’s multiple comparison test). (F) Cellular MDA content (*n*=3; one-way ANOVA followed by Tukey’s multiple comparison test). (G) PC12 cell viability in the four groups (*n*=3; one-way ANOVA followed by Tukey’s multiple comparison test). The values are presented as the mean ± SD. **P < 0.01, ***P < 0.001. ANOVA: Analysis of variance; GAPDH: glyceraldehyde 3-phosphate dehydrogenase; GPX4: glutathione peroxidase 4; MFI: mean fluorescence intensity; ns: not significant.

***Additional Figure 5:*** *TP53 manipulates ferroptosis induced by H2O2 and Fe in PC12 cells.*

Additional Figure 5 TP53 manipulates ferroptosis induced by H2O2 and Fe in PC12 cells.
(A) Transfection efficiency was evaluated by GFP. (B, C) Validation of the *Tp53* knockdown effect by Western blotting (*n*=3, unpaired *t*-test). (D, E) Validation of the effect of *Tp53* overexpression by Western blotting (*n*=3, unpaired *t*-test). (F) Immunofluorescence images of GPX4 in PC12 cells. (G) Immunofluorescence images of 4HNE in PC12 cells. (H, I) Quantitative statistics of GPX4 and 4HNE fluorescence (*n*=3; one-way analysis of variance followed by Tukey’s multiple comparison test). (J) GSH and MDA (K) content determination (*n*=3, one-way analysis of variance followed by Tukey’s multiple comparison test). The boxed border regions are enlarged. The values are presented as the mean ± SD. **P < 0.01, ***P < 0.001. GFP: Green fluorescent protein; GSH: glutathione; MDA: malondialdehyde.

***Additional Figure 6:*** *Proteins encoded by the top 10 candidate genes derived from Tp53 cleavage under targets and tagmentation analysis are subjected to molecular docking analysis with Slc7a11.*

Additional Figure 6 Proteins encoded by the top 10 candidate genes derived from Tp53 cleavage under targets and tagmentation analysis are subjected to molecular docking analysis with Slc7a11.

***Additional Table 1:*** *Information on the antibodies utilized for western blotting.*

***Additional Table 2:*** *Information on the antibodies utilized for immunofluorescence staining.*

***Additional Table 3:*** *Information on the primers used for quantitative reverse transcription-polymerase chain reaction.*

***Additional Table 4:*** *siRNA sequences and primer sequences for the plasmids.*

***Additional Table 5:*** *Information on the primers used for polymerase chain reaction.*

## Data Availability

*All relevant data are within the paper and its Additional files.*
